# Novel Methods for Analysing Bacterial Tracks Reveal Persistence in *Rhodobacter sphaeroides*

**DOI:** 10.1371/journal.pcbi.1003276

**Published:** 2013-10-24

**Authors:** Gabriel Rosser, Alexander G. Fletcher, David A. Wilkinson, Jennifer A. de Beyer, Christian A. Yates, Judith P. Armitage, Philip K. Maini, Ruth E. Baker

**Affiliations:** 1 Wolfson Centre for Mathematical Biology, Mathematical Institute, University of Oxford, Radcliffe Observatory Quarter, Oxford, United Kingdom; 2 Oxford Centre for Integrative Systems Biology and Department of Biochemistry, University of Oxford, Oxford, United Kingdom; University of British Columbia, Canada

## Abstract

Tracking bacteria using video microscopy is a powerful experimental approach to probe their motile behaviour. The trajectories obtained contain much information relating to the complex patterns of bacterial motility. However, methods for the quantitative analysis of such data are limited. Most swimming bacteria move in approximately straight lines, interspersed with random reorientation phases. It is therefore necessary to segment observed tracks into swimming and reorientation phases to extract useful statistics. We present novel robust analysis tools to discern these two phases in tracks. Our methods comprise a simple and effective protocol for removing spurious tracks from tracking datasets, followed by analysis based on a two-state hidden Markov model, taking advantage of the availability of mutant strains that exhibit swimming-only or reorientating-only motion to generate an empirical prior distribution. Using simulated tracks with varying levels of added noise, we validate our methods and compare them with an existing heuristic method. To our knowledge this is the first example of a systematic assessment of analysis methods in this field. The new methods are substantially more robust to noise and introduce less systematic bias than the heuristic method. We apply our methods to tracks obtained from the bacterial species *Rhodobacter sphaeroides* and *Escherichia coli*. Our results demonstrate that *R. sphaeroides* exhibits persistence over the course of a tumbling event, which is a novel result with important implications in the study of this and similar species.

## Author Summary

Many species of planktonic bacteria are able to propel themselves through a liquid medium by the use of one or more helical flagella. Commonly, the observed motile behaviour consists of a series of approximately straight-line movements, interspersed with random, approximately stationary, reorientation events. This phenomenon is of current interest as it is known to be linked to important bacterial processes such as pathogenicity and biofilm formation. An accepted experimental approach for studying bacterial motility in approximately indigenous conditions is the tracking of cells using a microscope. However, there are currently no validated methods for the analysis of such tracking data. In particular, the identification of reorientation phases, which is complicated by various sources of noise in the data, remains an open challenge. In this paper we present novel methods for analysing large bacterial tracking datasets. We assess the performance of our new methods using computational simulations, and show that they are more reliable than a previously published method. We proceed to analyse previously unpublished tracks from the bacterial species *Rhodobacter sphaeroides*, an emerging model organism in the field of bacterial motility, and *Escherichia coli*, a well-studied model bacterium. The analysis demonstrates the novel result that *R. sphaeroides* exhibits directional persistence over the course of a reorientation event.

**This is a** ***PLOS Computational Biology*** **Methods article.**

## Introduction

The motile behaviour of bacteria underlies many important aspects of their actions, including pathogenicity, foraging efficiency, and ability to form biofilms. The study of this phenomenon is therefore of biomedical and industrial importance, with implications in the control of disease [1] and biofouling [2]. Owing to their small size, bacteria inhabit a world of low Reynolds number, in which viscous forces dominate over inertia [3]. Rotational Brownian motion prevents them from swimming continuously in a straight line, hence many motile species such as the multiflagellate *Escherichia coli* move in a series of approximately straight ‘runs’, interspersed by reorientating ‘tumbles’ in a process known as taxis [4]. During a run, the flagellar motors in *E. coli* turn counter-clockwise, causing the helical flagella to form a rotating bundle that propels the cell forward. Tumbles are caused when one or more motors reverse their rotation, which disrupts the flagellar bundle and causes the cell to reorient randomly [4]. A related motile mechanism exists in the uniflagellate bacterium *Rhodobacter sphaeroides*, in which reorientations are, instead, effected by stopping the flagellar motor [5]. Upon ceasing to rotate, the single sub-polar flagellum [6] undergoes a change of conformation, leading to reorientation by a mechanism that is not yet well understood [7]. The biochemical pathways responsible for chemotaxis in *R. sphaeroides* are less well studied than those in *E. coli*, and are known to be more complex [8].

The tracking of bacterial cells, as imaged under a microscope, is a well-established experimental technique for investigating bacterial motility. Such studies have been used to gain biological insight in the case of *E. coli* [4], [9], *Pseudomonas putida* [10], *Rhizobium meliloti* [11], *Vibrio alginolyticus* [12] and *R. sphaeroides* [13]. A limitation of cell tracking is that a large number of tracks are required in order to ensure that any inferences drawn from observations are statistically representative of the population. Tracking experiments are therefore often laborious [14]. Earlier experiments involved tracking a single bacterium at a time, either in a fixed field of view [13], or by mechanically shifting the microscope stage to keep the cell in focus [4]. This approach suffers from subjective bias as the experimentalist is required to select which cells to track [14]. More recently, simultaneous multiple target tracking has enabled the measurement of tracks from all bacteria visible in the field of view at any given time [15]. This improves the efficiency of the experimental technique, allowing larger datasets to be obtained. It also reduces sampling bias, as all cells in the field of view are tracked. An experimental method related to tracking is differential dynamic microscopy (DDM), which enables the measurement of the distribution of swimming speeds and the fraction of motile cells in the observed population [16]. DDM records these statistics across very many bacteria, however it is an ensemble method and does not permit the measurement of the motile properties of individual bacteria.

Having acquired experimental tracking data, these must be analysed in order to extract quantities of interest. These include the distribution of swimming speeds [9], [13], [16]–[18], various measures of trajectory curvature [19], [20], turning angles [4], [10], the frequency of reorientations [18], [21], [22] and the extent of accumulation near a surface [23]. The ability to obtain such statistics permits quantitative investigations into the response of bacterial populations to environmental stimuli, in addition to cross-species comparisons and the true variability across a population. The analysis method used to extract statistics of motion from the raw data must be robust to errors in the tracking protocol, for example when cell trajectories intersect and the wrong paths are joined [4], and experimental noise such as errors in finding the centre of a cell. In order to identify reorientation events in bacterial tracks, both manual analysis [9], [22], [24] and heuristic arguments [4], [10], [18], [21], [25], [26] have been used. The former is prohibitively time-consuming when dealing with large datasets and is subjective. Automated heuristic methods may be effective in some cases, however it is important to validate such methods, and to avoid the introduction of systematic bias. To our knowledge, all existing heuristic methods require one or more threshold parameters to be specified. The process of selecting optimal threshold parameters may be automatable, as is the case with the method we use for comparison in our study, however this is not a straightforward task and in most cases no guidelines are given as to how to select optimal values for threshold quantities. For example, the method used by Amsler [21] requires the user to specify a threshold inter-frame angular velocity, above which the bacterium is said to be in a reorientation phase. Furthermore, of all the cited studies, only that of Alon et al. [18] includes an analysis of the sensitivity of the results to the various threshold parameters.

Here, we present novel methods for the automated, non-parametric analysis of large bacterial tracking datasets, based on a two-state model of the observed motion, which is compatible with any form of motile behaviour that is well-approximated by the run-and-stop or run-and-tumble models of motion. The data considered in this study are two-dimensional tracks, but the extension of the methods to three dimensions is straightforward. Our methods take advantage of the availability of non-chemotactic and non-motile mutants to gain empirical knowledge of the appearance of running and stopping phases in the observed motion. The methods are based on a modification to the hidden Markov model (HMM), and are applicable to any bacterial species where such mutants exist and sufficiently long reorientation events are discernible using video microscopy. In addition, we suggest a straightforward method that is applicable in the absence of a non-motile mutant. We use a simulation study to assess the performance of the new methods, and compare them with a heuristic approach. To our knowledge such a systematic comparison of methods has not previously been attempted in this field. In order to demonstrate the wide application of our methods, we apply them to analyse novel *R. sphaeroides* and *E. coli* datasets, acquired using a recently developed tracking protocol [27]. We show how our new methods enable us to determine the previously unreported distribution of angle changes during a reorientation in *R. sphaeroides*, amongst other characteristics of the observed motion.

## Results

**Figure 1:**
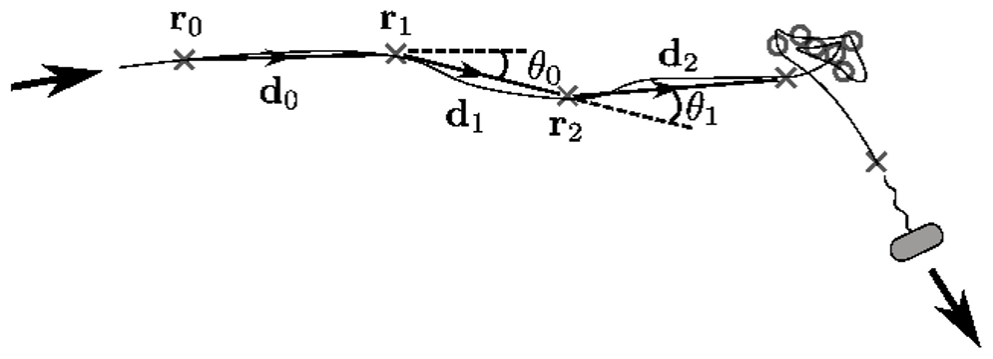
Data representation in a track. The thin black line represents the continuous trajectory of a cell. Crosses and circles denote running and stopping phases, respectively, and represent locations at which the position of the cell is recorded, separated by a constant time interval 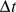. Dashed black lines and notation illustrate the mathematical representation of the track.

Bacterial tracks of *R. sphaeroides* and *E. coli* were acquired as detailed in Materials and Methods. Figure 1 shows a cartoon illustration of a single track. A bacterium swims in an approximately straight line, enters an approximately stationary stopped phase for some time, then swims off in a new direction. The crosses indicate observations made of the cell centroid at regular intervals, 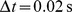 (videos are typically captured at 50 frames per second). The primary focus of this study is the identification of stops as illustrated in Figure 1. This task is complicated by various sources of noise in the data. These include: (i) uncertainty in the position of the centroid of a cell in each image that may cause a track to appear jagged, for example when the cell body rotates whilst swimming; (ii) Brownian buffeting that may also cause departures from straight-line swimming, and lead to stops that are not perfectly stationary; (iii) tracking errors caused by incorrectly linking cells between consecutive frames, or by the disappearance of a cell for one or more frames, that may affect the appearance of a track. The identification of stopping phases in tracks is therefore a challenging process.

Each track generated by the tracking procedure is represented in the form 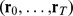, where 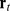 designates a two-dimensional position vector at time 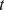, and the number of frames in the track is given by 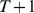. Note that 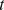 is considered a discrete quantity throughout, as time is measured in numbers of frames. In characterising running and stopping phases, we are concerned not with the positions of cells in each frame, but with the motion of cells between consecutive frames. The information of interest is thus the transitions between consecutive position vectors within a track. These form a list of displacement vectors, 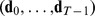 with 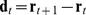. The framewise speed is defined as the observed speed of travel between two consecutive frames, 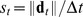, where 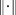 denotes the Euclidean norm. The angle changes 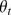 between consecutive vectors, henceforth called framewise angle changes, are defined so that 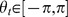 gives the difference in polar angle between 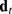 and 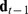.

We assume a two-state model of cell motility, in which each displacement vector, 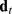, corresponds to either a running or stopping state. The underlying state at time 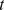 is denoted 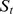, where we use the convention throughout that 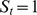 corresponds to a stop and 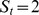 corresponds to a run, hence for each track a state vector 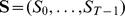 describes the sequence of states. We wish to assign to each displacement vector a probability of being in a running phase, 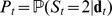. Note that, since we assume a two-state model, we have 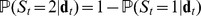.

We use our methods to analyse tracking data from *R. sphaeroides* and *E. coli*. In each case, data are obtained from three strains: a wildtype strain, which undergoes discrete running and reorientation phases, a non-chemotactic strain, which is always in the running phase and exhibits no reorientation events, and a non-motile strain, which is unable to propel itself.

### Analysis methods

There is no well-established gold standard for identifying reorientation events in bacterial tracks; indeed several tracking studies make no attempt to extract quantitative information about the reorientation events in tracks [15], [28], [29], while others use ensemble measures such as angular velocity as a proxy for the rate of reorientation [24], [30]. Various heuristic methods requiring the specification of one or more threshold parameters have been used in tracking studies in bacteria (see the related discussion in Introduction). In this study we compare our methods with that of Taboada et al. [25], which is sufficiently versatile to apply to our current data with little modification. This is henceforth denoted the heuristic method. The focus of the present work is the development and validation of our novel analysis methods, however we note that several other heuristic methods mentioned above may be applicable providing it is possible to automatically optimise the various threshold parameters involved. We do not consider these further as a complete survey of methods is beyond the scope of this paper.

We now describe the heuristic method and the two novel analysis methods considered throughout the rest of this work. In addition, we describe a ‘post-processing’ heuristic that can improve the performance of all of the methods and is particularly effective when combined with the heuristic method.

#### Heuristic method

The intuitive approach used by Taboada et al. [25] is to define a cutoff speed parameter 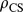 and denote each transition as a run if the framewise speed is greater than 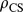, so that
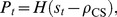
(1)where 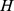 denotes the Heaviside function. The key assumption underlying the heuristic method is that there is a substantial difference between the distribution of framewise speeds observed during runs and stops. The value of 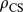 should be selected to maximise the number of correctly inferred transitions. We approach the problem of optimising 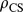 by computing the observed framewise speeds for the non-chemotactic and non-motile strains. We estimate the true probability density function (pdf) of framewise speeds in each case using a kernel density estimate (KDE). We then take 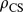 to be the point at which the two pdfs intersect. Note that this method is not guaranteed to minimise the crossover region between the two pdfs, but is a reasonable approximation. The implementation of the KDE by Botev et al. [31] used in this study represents a non-parametric method for determining the kernel bandwidth, thus avoiding the need to select an arbitrary histogram bin width.

A problem associated with the heuristic method lies in the choice of the parameter 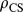. If there is any overlap between the framewise speed distribution for run phases and stop phases, due to the effects of noise and/or population heterogeneity, then this approach will cause spurious inference in the crossover region. Nevertheless, this approach is acceptable if the level of noise in the data is such that the distributions are well separated.

#### Hidden Markov model (HMM) methods

Our novel approach to the analysis of bacterial tracks utilises a state space model with an empirical prior to infer the state probabilities. We apply a HMM to the observed data. For brevity, we assume familiarity with the basic HMM; a detailed tutorial is given by Rabiner [32]. Details of the numerical implementation of the HMM are given by Press et al. [33]; the notation used here is the same as in this reference. Methods based on the HMM have previously been successfully applied to data from DNA looping and single particle tracking experiments [34], [35]. The application of the HMM to the analysis of bacterial tracks requires a modification to the standard HMM formulation, similar to those described by Beausang and Nelson [34] in their study of DNA looping dynamics.

We assume that the observed motion between sampling points in each track arises from a hidden two-state Markov chain, where the states correspond to running and stopping phases. We denote the transition matrix by 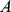, with entries 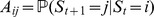, where 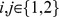 as previously discussed. In the absence of any chemoattractant or chemorepellent concentration gradient, we assume that the Markov chain is homogeneous, meaning that the probability of switching from a run to a stop (or *vice versa*) is independent of time and space. The initial state probabilities are denoted by 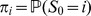. The continuous observation pdf is denoted by
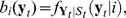
(2)which gives the pdf of observing the datum 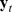 at time 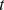, conditional on the system being in state 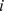. The observation pdf gives the prior probability density of observing a particular movement, conditional on the cell's state. It is obtained empirically from novel experimental data of non-motile and non-chemotactic strains. Full details on the form of the observation pdf are given below.

Implementation of the HMM requires the computation of two quantities, the forward and backward estimates. These are defined by
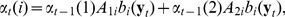
(3)and
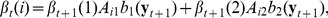
(4)respectively for 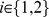. Note that 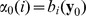, and 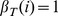 by convention. These two quantities may be used to define the probability of the state at time 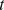 being a run:
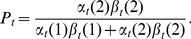
(5)We consider two variants of the HMM-based analysis approach, which differ in the way in which the observed data 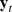 are represented. We first describe the full HMM method, in which both framewise speeds and angle changes are considered. We then describe a simplified variant, in which only speed data are used.

The full HMM approach uses both the framewise speed and the framewise angle as the observable data, 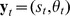. Since we determine the observation pdf empirically, the only free parameters in the model are the state transition probabilities 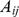. We assume that switching from state 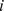 to state 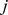 occurs with a characteristic time 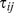. The transition matrix is therefore given by
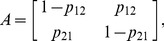
(6)where 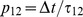 and 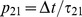, which are interpreted as the probability of stop-to-run and run-to-stop transitions, respectively. In order to ensure that both of these quantities are in the range 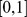, the camera frame rate must be set sufficiently high, so that 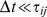.

The observation pdf encodes our prior knowledge of the distribution of framewise speeds in the running and stopping states. We assume that the non-chemotactic mutant swims in the same way as a wildtype bacterium in a running phase, and that the motion of the non-motile mutant is similar to that of a wildtype bacterium in a stopping phase. Experimental justification of this assumption is given in *Analysis of experimental data*. The standard HMM formulation requires that the observation pdf is independent of all previous states: the single subscript in the observation pdf, 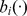, refers to the current state. In order to incorporate angular information in our analysis, we must relax this requirement, so that the observation pdf may depend on both the current state and preceding states [34]. This modification is necessary because tracking cell movements is less accurate when the cell is in a stopped phase than when the cell is running, due to the smaller motions involved. As a result, the computed framewise angle changes contain a significant source of error. During a stop-to-run transition, the framewise angle change observed may differ a great deal from that predicted based on the previous, noisy observed direction of motion during the stop. Use of the standard HMM formulation could then lead to the stop-to-run transition being incorrectly classified as a stop-to-stop transition on the basis of an apparently large framewise angle change. We therefore define a modified observation pdf to account for this source of error, in which there is a dependence on the previous state, in addition to the current state, given by
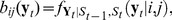
(7)where 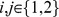. The modified observation pdf 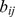 does not break the Markov property of the process, since the dependence is limited to the current and previous states. Instead, using 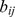 allows us to take experimental technicalities into account. For a further example of such a modification, see the study by Beausang and Nelson [34]. No modification is required to the transition matrix.

Including this modification in the conventional HMM formulation, equation (3) becomes
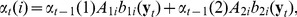
(8)and the analogous expression for equation (4) is given by
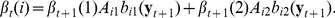
(9)We assume independence of speed and angular distributions so that 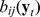 is separable,
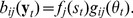
(10)This simplifying assumption is necessary as we do not have sufficient data in the present study to estimate a joint distribution accurately. This may be possible in future studies; the modification of the current methods to use such a distribution is straightforward. Plots showing the form of the noisy joint distributions are provided in Figures S1, S2, S3, S4 for reference. In equation (10) the speed component, 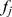, is independent of the previous state whereas the angular component, 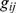, is not. Estimates for these components are obtained from experimental data acquired from mutant strains. The speed component is equal to the KDE of observed framewise speeds in the non-motile mutant when 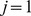 and the non-chemotactic mutant when 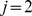. The angular component is equal to the KDE of observed framewise angle changes in the non-motile mutant when 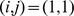, 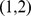, or 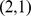, and the non-chemotactic mutant when 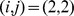. Note that the modification is required to take into account the use of the non-motile distribution for 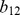, as discussed above.

In order to implement the algorithm described above, one final detail is required. There is no guarantee that the empirical estimate for the speed component of the observation pdf is non-zero for all observed framewise speeds in the wildtype dataset, since those tracks are effectively hidden when we generate the empirical priors. It is important to avoid a situation in which the observation pdf is numerically equivalent to zero, which occurs when 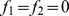, since this causes the algorithm to fail by declaring that the track is in neither the running nor the stopping state, hence breaking the two-state assumption. This could occur if tracks in the wildtype dataset contain some high framewise speeds, relative to the non-chemotactic dataset. Such an eventuality is avoided by adding a small numerical constant to the speed component to ensure it is non-zero for all permissible speeds (see Materials and Methods for an explanation of why there is an upper bound to the permissible framewise speeds).

In the speed-only model, we consider only the framewise speed as the observable data. This is achieved by a straightforward modification to the full model, in which we impose the circular uniform distribution on the angular component of the observation pdf, 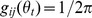.

**Figure 2:**
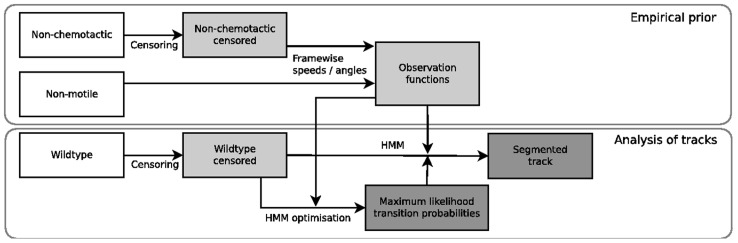
Flow diagram of the stages involved in analysing the experimental tracking data. White boxes represent the raw datasets. The non-chemotactic and wildtype data are first censored to remove spurious tracks, as described in Materials and Methods. The two mutant strains are then used to generate an empirical prior, in the form of the observation functions. The empirical prior is used when analysing the wildtype dataset, in order to find the MLE of the transition probabilities and finally segment the track into discrete states by computing the state sequence, 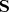.

The two components describing the HMM are the observation pdf, 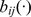, and the transition probabilities, 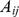. The observation pdf is independently determined from observations of non-chemotactic and non-motile strains, and 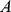 is specified by the two parameters in equation (6), namely 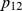 and 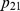. It is possible to obtain a maximum likelihood estimate (MLE) [33] of these free parameters by maximising the likelihood of the data given the model, defined by
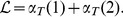
(11)We may use the MLE to estimate the dwell times, 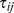, providing that the limitation 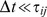 is respected. Das et al. use a Markov chain Monte Carlo scheme to find the MLE of their rate parameters in a similar application to that described here [35]. In our case, the negative log-likelihood surface is always found to be smooth, with a unique minimum (data not shown), so that a deterministic optimisation routine is more computationally efficient. We use a MATLAB implementation of the trust-region-reflective algorithm to carry out a constrained numerical optimisation of the negative log-likelihood [36]. The function to be minimised is defined by
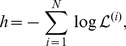
(12)where 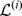 denotes the likelihood of the data from the 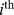 track, and 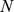 is the total number of tracks in the dataset. As the likelihood is a function of 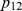 and 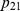, the minimisation is carried out over a two-dimensional vector space. We estimate 95% two-tailed confidence intervals for our MLE of 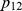 and 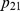 using the basic bootstrap method [37], with 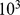 bootstrap iterations. The summation in equation (12) pools the results from all of the tracks in the censored dataset, so that the MLE is an ensemble quantity. It is possible, in principle, to maximise the likelihood over each individual track, however the performance of this approach is poor when dealing with short tracks (data not shown). The optimised parameters are subsequently used to compute the run probabilities using equations (3)–(5). We summarise the analysis pipeline in Figure 2.

#### Post-processing

**Figure 3:**
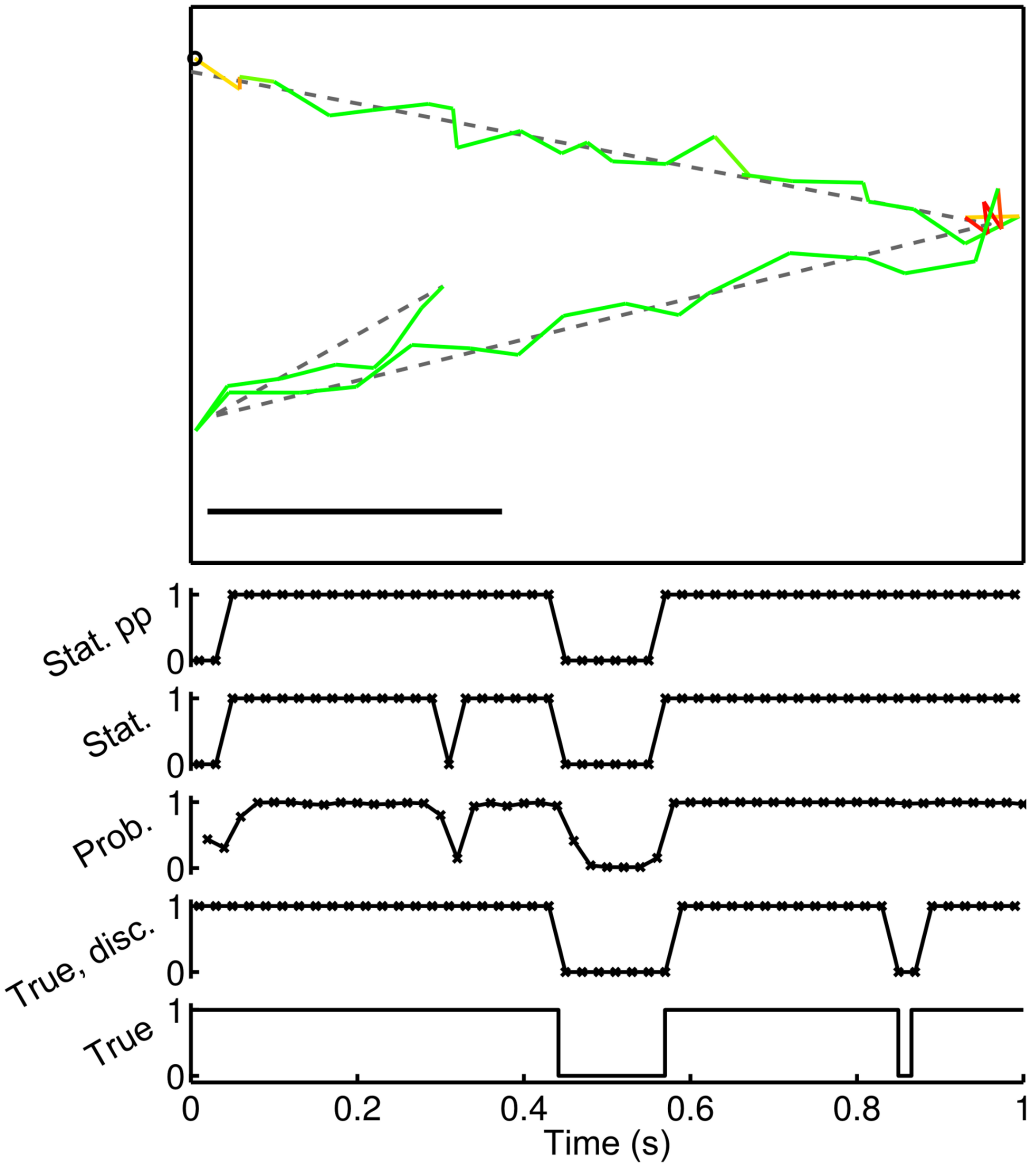
An illustration of the output of the analysis methods, post-processing and comparison with the true underlying state for a simulated track. The upper panel shows the simulated track; the black circle shows the start point, dashed lines indicate the true underlying motion, and coloured lines indicate the observed motion after the addition of noise. Colours correspond to run probabilities, as inferred by the full HMM method, with a colour map that varies between green, denoting a run, and red, denoting a stop. The scale bar is 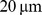 in length. The lower plots show (from bottom to top) the true underlying state, before and after discretisation, the run probabilities, and the run status, before and after post-processing. Crosses indicate sample points.

Each of the analysis methods returns a vector for each track, containing the probability of a run between each observation point, 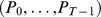. In the case of the heuristic method, every value is equal to 1 or 0, whereas the HMM methods return values in 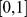. In the latter case, we round all values to the nearest integer (0 or 1). The resulting vector can be considered to represent the run status (as opposed to run probability). This transformation is always carried out on the run probabilities computed using the HMM-based methods. In the case of the heuristic method, there is no distinction between the two properties. The difference between run probability and run status is illustrated in Figure 3.

An additional heuristic step may be applied to the run status vector of each track, which smooths the inferred state path between the running and stopped phases. We define a run persistence parameter, 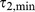, and a stop persistence parameter, 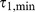, which correspond to the minimum permissible duration of running and stopped phases, respectively. Running phases that have durations shorter than 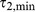 are relabelled, and likewise for stopped phases shorter than 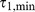, so that the whole track has a valid run status. Details of the implementation are given in Materials and Methods. These minimum permissible duration parameters should be selected appropriately for the system being studied and the parameters of the experimental protocol. For example, if the sampling rate is very rapid relative to the mean stopping duration, this would suggest that a large value of 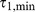 may be appropriate. We do not consider the process of selecting these parameters further as they are an optional addition to our analysis method; the main purpose of their inclusion in this study is to show how they may improve the output of the heuristic approach (see the following simulation study).

### Simulation study of analysis methods

Prior to applying the heuristic method and our two novel methods to experimental data, we must evaluate and compare their ability to correctly infer stop phases in tracks affected by various levels of noise. A traditional means of evaluating this performance is to compare with the results of manual assignment of stopped phases in real tracks. This approach suffers from several key drawbacks, however. Manual tracking is a time-consuming and often difficult process; the stopped phases in microscope videos are by no means easy to discern unambiguously by eye. In addition, manual assessment of tracks is unavoidably subjective.

Here we use an alternative approach to manual analysis: a simulation study. This is a common means of assessing the performance of automated analysis methods [34], [35], [38]. We assume that experimentally-obtained wildtype tracks are the result of a run and stop velocity jump process [39]. Cells in the running phase travel in straight lines with a constant speed drawn from a Weibull distribution that closely approximates the observed non-chemotactic running speed distribution. After a random, exponentially distributed time interval with mean 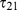, cells enter a stopping phase and their speed is set to zero. Cells stop for a random period of time, exponentially distributed with mean 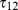, after which they switch to the running phase again with a new, Weibull distributed run speed. A new direction of travel is drawn at each reorientation event from the circular uniform distribution. We also simulate tracks describing the non-chemotactic mutant, in which no reorientation events occur, and the non-motile mutant, which is always in the stopped state. We define the sampling interval to be 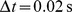 to match the frame capture rate of the microscope used to obtain experimental movies. We simulate 500 tracks for 250 frames each using the parameter values 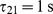 and 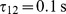. These mean duration values are in close agreement with previous studies of *E. coli* [4], while the remaining simulation parameters have been chosen to match the experimental protocol used to acquire tracks in this study (see Materials and Methods).

We include a simplified model of the noise in the system by adding a normally distributed perturbation to each coordinate of every recorded position in a track, with zero mean and variance equal to 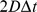, where 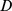 is varied to modulate the level of noise applied to the system. A random selection of simulated tracks with varying levels of noise are shown in Figure S5. We note that the use of uncorrelated Gaussian noise to simulate the type of noise exhibited in real experimental data may be an oversimplification, however the nature of the noise present in such cases is unknown and beyond the scope of this study. The true underlying state sequence in the simulations, which is continuous in time, is recorded for later comparison with the state inferred by the analysis methods. In carrying out the steps required to analyse the simulated datasets and compare their performance, we attempt to mimic as closely as possible the process that we use when analysing real data (see Figure 2). We infer the values of all model parameters based on the three simulated datasets; none of the parameters of the true underlying processes are known to the analysis methods.

Before commencing the simulation study, we verify that the methods do not produce spurious results when applied to tracks generated from an incompatible underlying model of motion. This test is carried out by analysing tracks from a non-chemotactic simulated dataset. Such tracks contain no stops; the aim of this initial test is to ensure that the analysis methods do not infer stopping phases falsely. In practice, we find that the optimisation routine fails to find a MLE for the transition rate parameters because the negative log-likelihood is independent of the parameter 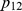 (see Figure S6 and Text S1 for details). This indicates that the HMM-based methods cannot be applied blindly to tracks that contain no stops.

**Figure 4:**
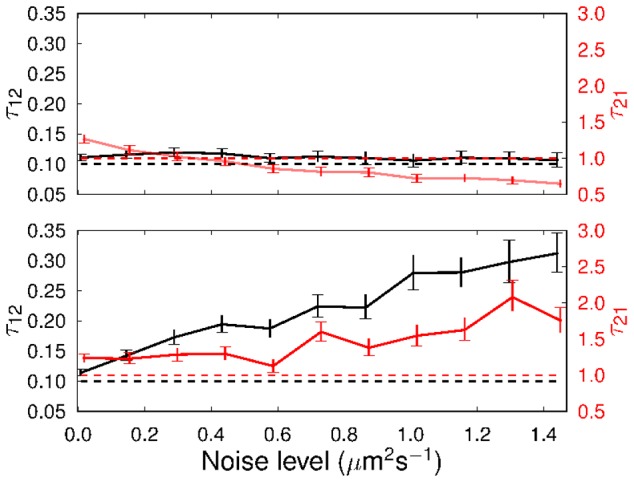
MLE mean durations and 95% confidence intervals, 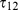 (black) and 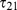 (red), computed with simulated tracks by minimising the negative log-likelihood. (Top plot) HMM full; (bottom plot) HMM speed-only. Dashed lines indicate the true values used in the simulation.

Figure 4 illustrates the MLE values and 95% two-tailed confidence intervals of the mean running and stopping durations, 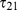 and 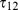, respectively, for a range of values of the noise level, 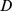. When the level of added noise is low, the two parameters are estimated correctly by both methods. The MLE value of 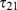 is overestimated by around 20% by both methods in the absence of noise. In the case of the full HMM method, the MLE value decreases with increasing noise level, which initially causes the estimate to become more accurate. At the highest noise level considered here, the MLE 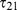 is around 60% of the true value. In contrast, the speed-only method MLE 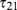 increases with noise level. At the highest noise level, the MLE is around double the true value. The full method estimates the value of 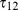 accurately throughout the range of noise levels considered, whereas the speed-only method increasingly overestimates the same parameter as the noise level increases. At the highest noise level, the speed-only MLE 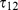 is around threefold greater than the true value. Since the noise model incorporated in our simulations may differ from the sources of noise in the experimental tracks, the precise quantification of the error in the MLE is not of real interest here. However, this result suggests that parameters estimated from highly noisy data may be unreliable, and that the full HMM method generally provides better estimates.

All of the analysis methods output a run status vector for each track, which is discrete in time. The true underlying state path is, by contrast, continuous in time. In order to facilitate a comparison between the inferred state sequence and the ground truth, we discretise the ground truth over intervals of duration 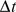. Any such interval that contains part of a stop phase is designated a stop in the discretised true state sequence. The inferred state sequence is a series of stopping phases and running phases, with the convention that an inferred stop corresponds to a positive result. A false positive (FP) therefore corresponds to an inferred stopping phase where none is present in the true underlying state sequence, while a false negative (FN) corresponds to an inferred running phase where none is present in the true underlying state sequence. Figure 3 illustrates this; compare the true, discretised run status with the inferred run status. There are several discrepancies. A stop lasting two frames is inferred at the start of the track, where none is present in the true state. This is a FP; there is another at around 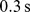. Conversely, at approximately 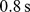 a true stopping event is missed by the analysis method. This is a FN. As noted previously, the application of the post-processing method with 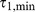 and 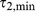 both greater than one corrects the second FP. For each level of added noise, we compute the mean rate of FPs and FNs as the ratio of the total number of FPs and FNs to the total number of actual stop events in the true underlying state. This is computed as the average over all tracks in the simulated dataset.

**Figure 5:**
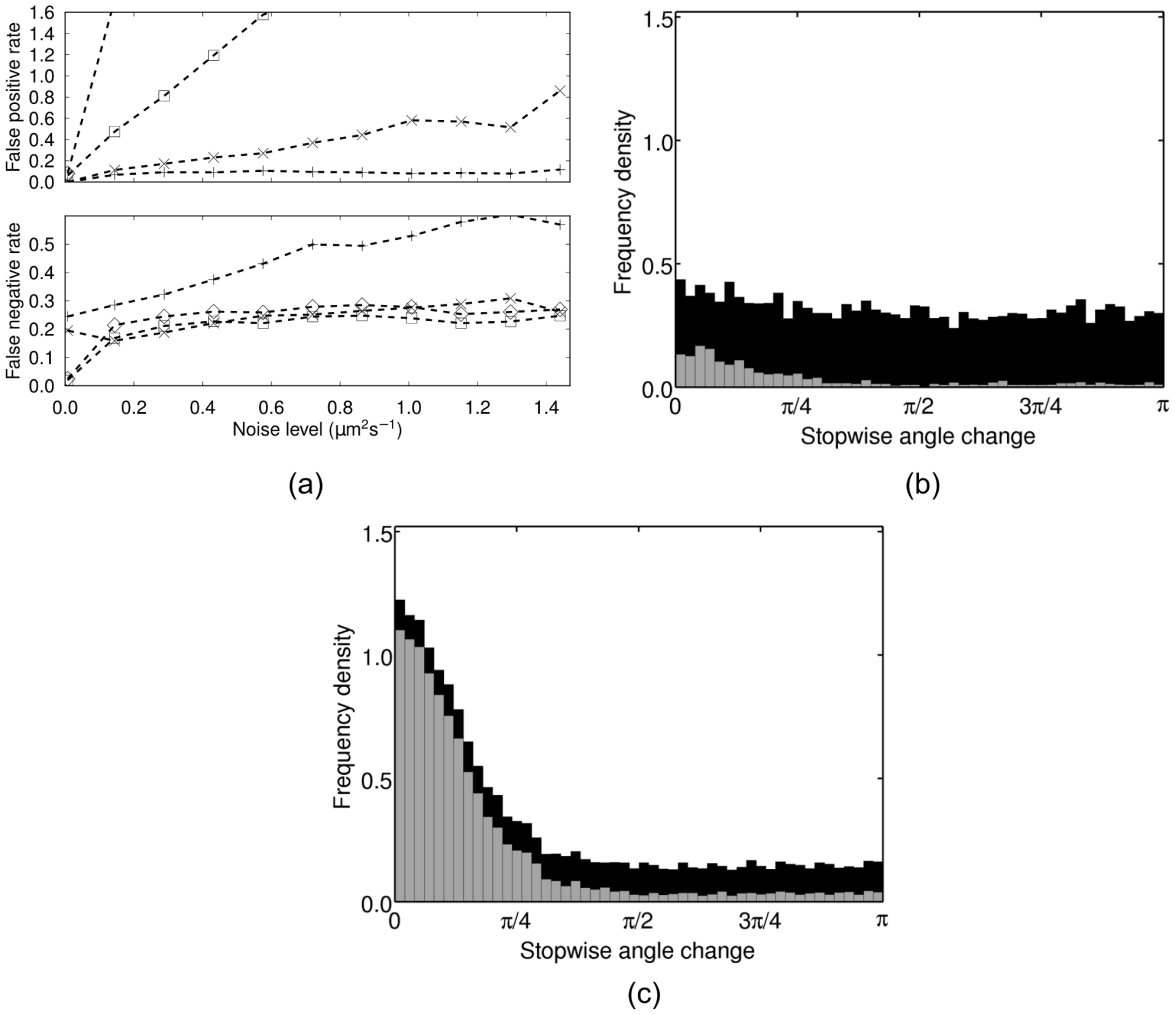
Assessing the performance of the analysis methods using simulated data. (a) Mean FP and FN rate per simulated track at different levels of additive noise. (◊) heuristic, no post-processing; (□) heuristic with post-processing; (+) HMM speed-only, no post-processing; (×) HMM full, no post-processing. (b) and (c) Histograms of the inferred stopwise angle changes computed using the full HMM method (b) and the heuristic method (c) on the simulated dataset with 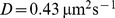. Black bars show data for all inferred stops, grey bars show which of these are due to FPs. The results are similar when the speed-only method is used, or if post-processing is applied.

Figure 5(a) shows the mean FP and FN rates produced by the three analysis methods. In the case of the heuristic method, we test the results with and without post-processing with 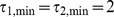. The application of post-processing made no significant difference to the results from the HMM methods (data not shown). A FP rate of one means that the average number of false stops equals the number of true stops, while a FP rate of zero indicates that no FPs are observed. The heuristic method is highly sensitive to low levels of noise, generating significantly higher FP rates than the methods based on the HMM. The heuristic FP rate is reduced somewhat by the application of post-processing, however it still remains significantly higher than either of the HMM methods. The full HMM method has a higher FP rate than the speed-only method, though the discrepancy only becomes large when 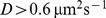. The speed-only method has an approximately constant low FP rate throughout the full range of noise levels considered here. In contrast, the speed-only method generates the largest FN rate, with the full HMM and heuristic methods exhibiting a similar, lower FN rate. These results suggest that the full HMM method is better able to identify stops, with the disadvantage that it is also more sensitive to noise and more prone to false positives. On the other hand, the speed-only method detects fewer stops, but makes fewer false declarations.

We further assess the accuracy of the HMM methods in Figure 5(b) by plotting the histogram of all inferred angle changes over the course of a stopping phase (henceforth denoted stopwise angle changes), overlaid with the histogram of stopwise angle changes due to FPs. We use a simulated dataset with an intermediate level of additive noise (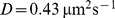) for this purpose, as this is similar to the value of the translational diffusion coefficient estimated from the experimental data (approximately 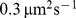; see Figure S10 and Text S1). The result changes very little for noise levels up to 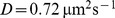 (data not shown). The true underlying distribution of stopwise angle changes is uniform. This figure shows that FPs tend to produce small stopwise angle changes, which introduces some bias into the process. However, the number of FPs is low and the bias is not significant over a range of intermediate noise levels. As Figure 5(c) illustrates, the bias is significantly higher when the heuristic method is used. This study indicates that the novel HMM methods developed here represent a demonstrable improvement over the heuristic method for the identification of stopping phases in tracks. In particular, the level of FPs and degree of systematic bias introduced by the heuristic method are unacceptable, as they would lead us to draw erroneous conclusions from our data.

### Analysis of experimental tracking data

In this section, we restrict our attention to the HMM-based methods, as the simulation study demonstrated that the FP level is unacceptable using the heuristic method when even low levels of noise are present. Our aim is to demonstrate the broad relevance of our methods to various species of motile bacteria. To this end, we consider two novel datasets, obtained for *R. sphaeroides* and *E. coli* as described in Materials and Methods. Results from the analysis of *R. sphaeroides* are shown in full. Many previous studies have considered the motile behaviour of *E. coli* [4], [9], [40], therefore for reasons of space we only present the main results from this dataset.

**Figure 6:**
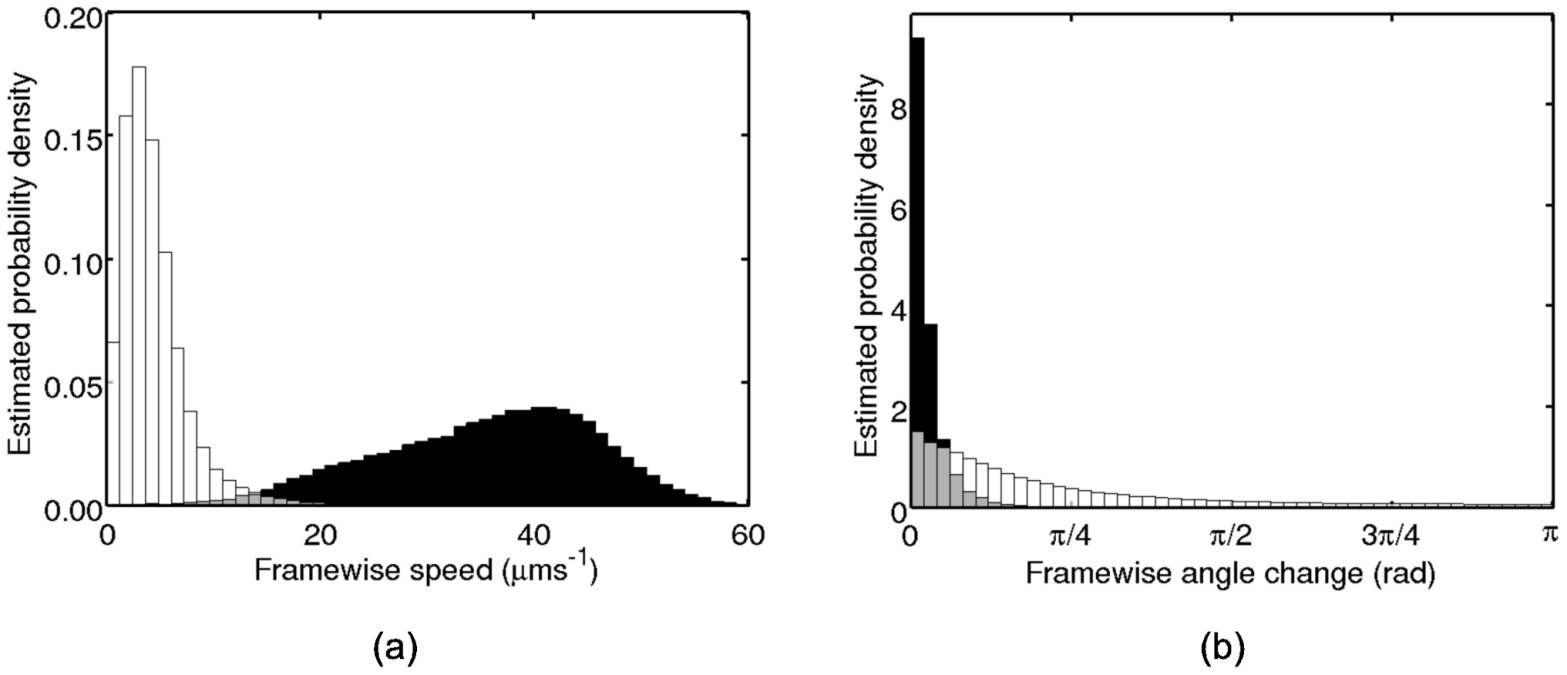
Observed distributions extracted from the non-motile (white bars) and non-chemotactic (black bars) *R. sphaeroides* mutants, after censoring. Grey bars denote overlapping regions. (a) Framewise speeds. (b) Framewise angle changes.

We use the non-chemotactic and non-motile datasets to form the empirical prior in the HMM-based methods. This is achieved by computing the framewise speeds and angle changes in both cases and applying the KDE to estimate the observation pdfs, as described previously. The emprirical prior for the *R. sphaeroides* dataset is plotted in Figure 6.

The inferred maximum likelihood parameters are shown in Table 1 along with other values reported in the literature. Our simulation study indicated that both HMM-based methods generated MLEs that differed from the true values, with the speed-only method likely to overestimate both 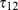 and 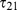 and the accuracy of the full method depending on the level of noise. This is borne out in our analysis, with the speed-only method generating larger MLEs for both *R. sphaeroides* and *E. coli*. The discrepancy between the two methods in the inferred transition rates is thus an indication that our estimates of the transition rates should be treated with caution.

**Table 1:** Mean duration of running and stopped states.

Reference	Species	Method	*τ*_12_ (s)	*τ*_21_ (s)
[4]	*E. coli*	Single cell tracking	0.14±0.19	0.86±1.18
[41]	*R. sphaeroides*	Tethered cell	0.27	1.69
[42]	*R. sphaeroides*	Tethered cell	0.66±1.01	3.23
[62]	*R. sphaeroides*	Tethered cell	1.04±3.18	4.54
This study	*R. sphaeroides*	Tracking (full)	0.40±0.02	1.16±0.06
This study	*R. sphaeroides*	Tracking (speed-only)	0.50±0.02	1.59±0.08
This study	*E. coli*	Tracking (full)	0.19±0.01	0.35±0.01
This study	*E. coli*	Tracking (speed-only)	0.31±0.01	0.53±0.02

Summarised literature values of transition rates between the running and stopped states in *R. sphaeroides* and *E. coli*. Standard deviations are given where they are available; note that standard deviations provided for the analysis methods refer to the optimisation procedure rather than the difference between individual tracks. The terms ‘full’ and ‘speed-only’ refer to the HMM method used to analyse the data.

A wide range of transition rates have been recorded in the studies cited in Table 1, despite the superficially similar experimental protocols. A few of the many possible explanations include the use of different wildtype strains, small differences in the composition of the motility buffer, and differences in the analysis methods. Comparing with our results, we see that the inferred value of the mean stop duration in *R. sphaeroides* is in reasonable agreement with the findings of Berry et al. [41]. The results suggest that running phases occur for a shorter mean duration in our datasets than those of Brown [42] or Packer et al. [43], as indicated by the smaller value of 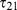. Results for *E. coli* are in reasonable agreement with those of Berg and Brown [4]. The tethered cell and tracking protocols differ a great deal, as observed by Poole and coworkers [13], who noted that the use of antibody to tether *R. sphaeroides* to a microscope slide by their flagella substantially reduced their rotation speed and decreased the number of observed stops. This is consistent with our findings, as we estimate a smaller value for 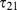, corresponding to shorter runs and an increased number of stopping phases.

Furthermore, we note that our MLEs are computed for pooled data, so that individual variations between tracks are averaged over an entire dataset. There is considerable heterogeneity in switching rates within a bacterial population [43]. However, considering each track separately would result in insufficient data being available for shorter tracks, or those containing no run-stop-run transitions, so we do not consider that problem here. It is for this reason that the estimate of the error in the MLEs is low in comparison with the other results cited; this is because we use bootstrapping of our ensemble sample to generate this estimate (see Materials and Methods for details). The error estimated in our study is therefore a reflection of the nature of the negative log-likelihood surface close to the MLE, rather than an estimate of the deviation between individual tracks. It may be possible to investigate population heterogeneity by applying the HMM-based methods to individual tracks obtained using single-cell tracking methods, as these tracks are generally longer.

**Figure 7:**
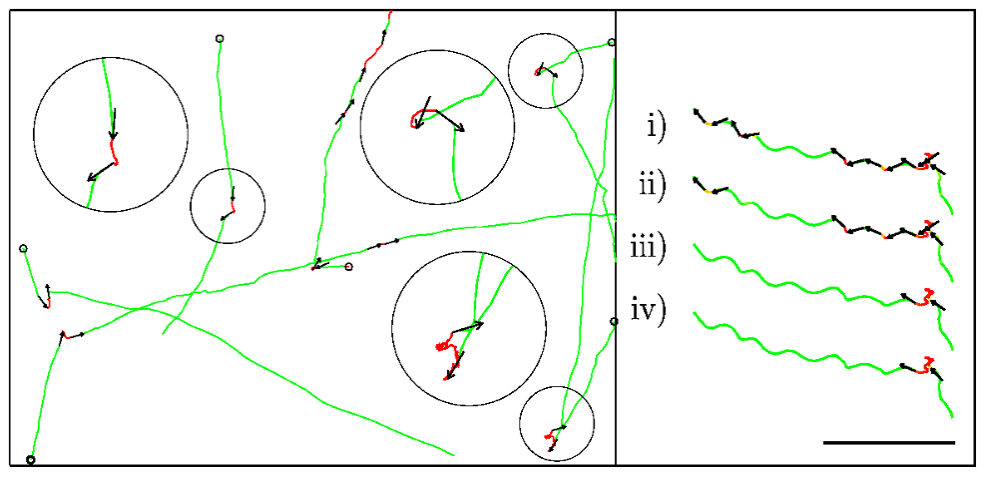
Manual inspection of *R. sphaeroides* tracks to assess the performance of the analysis methods. (Left) A selection of tracks that were manually verified to contain stopping phases correctly identified by the speed-only HMM method. Green indicates a running phase, red indicates a stopping phase, small circles indicate the starting position of the track, and pairs of arrows show the direction of travel of the bacterium immediately prior to and after a stop. Larger circles indicate regions of the track that have been expanded in the nearby inset plots. (Right) A track from a bacterium swimming in a helical trajectory, as analysed by (i) full HMM, (ii) speed-only HMM, (iii) full HMM with post-processing, and (iv) speed-only HMM with post-processing. The black bar is 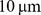 long, otherwise the plot is interpreted as for the left-hand side.

In contrast with our simulation study, we have no ground truth with which to compare the result of the analysis of the experimental datasets. Nevertheless, a manual inspection of the inferred state sequence of tracks readily identifies some tracks in which the analysis appears to be successful, in addition to some tracks in which the inferred state sequence is unrealistic. A selection of wildtype *R. sphaeroides* tracks in which the analysis has been manually identified as successful is shown in Figure 7 (left panel). Several well-defined stopping regions within the tracks have been expanded for greater clarity. Note that, although the speed-only HMM method was used to compute the run probabilities in this figure, the results for these tracks are almost indistinguishable when the full HMM method is used. The track shown in Figure 7 (right panel) arises from a bacterium swimming slowly in an exaggerated helical trajectory, and appears to contain a single genuine stopping event. Both analysis methods incorrectly identify several of the helical turns as stopping phases, leading to an unrealistically rapidly oscillating state sequence. Application of post-processing to either HMM analysis method circumvents this issue. The presence of such a track in the censored dataset motivated a manual examination of all tracks exhibiting either high median curvature or containing a large number of inferred stopping phases. This indicated that, of the 2780 tracks included in the wildtype dataset, fewer than five are clearly identifiable as highly tortuous. Any effects from this minority of tracks, after pooling all analysed data, will be insignificant. A similar outcome is observed in *E. coli*, although the proportion of tortuous tracks appears to be higher (data not shown). We provide the analogous plot to Figure 7 for *E. coli* in Figure S11.

**Figure 8:**
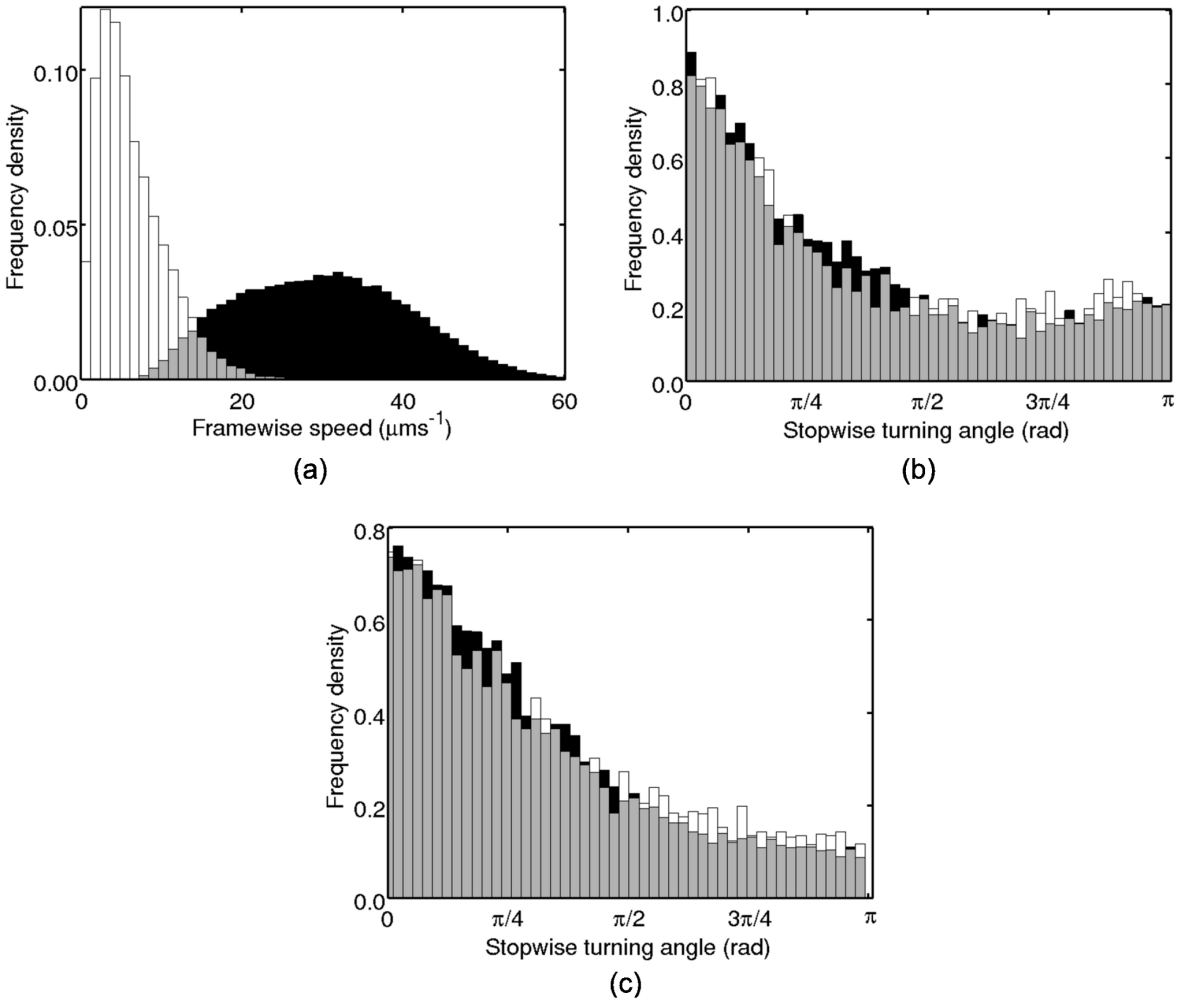
Characteristics of the motile behaviour of wildtype *R. sphaeroides* extracted using the HMM-based analysis methods. (a) Observed distribution of framewise speeds in the running (black bars) and stopping states (white bars), computed using the full HMM method without post-processing. Application of post-processing and/or using the speed-only method makes no significant difference to the results. (b) Observed distribution of absolute stopwise angle changes computed using the full (black bars) and speed-only HMM method (white bars) without post-processing. Application of post-processing makes no significant difference to the results. (c) As (b), but for *E. coli*. In all plots, grey bars denote overlapping regions.

In Figure 8(a) we provide a verification of our assumption that wildtype bacterial motility in *R. sphaeroides* may be approximated as consisting of runs, which are equivalent to those of the non-chemotactic strain, and stops, equivalent to the behaviour of the non-motile strain. This figure shows the observed distribution of framewise speeds in the phases identified as running and stopping by the analysis methods. These are qualitatively similar to those in Figure 6, suggesting that the form of our empirical prior is appropriate. Furthermore, the similarity of the distributions estimated by the speed-only and full methods indicate that the two methods are in close agreement.

Figures 8(b) and 8(c) show the estimated distribution of absolute stopwise angle changes in *R. sphaeroides* and *E. coli*, respectively, as computed using the speed-only and full HMM methods without post-processing. Plotting angles rather than absolute angles does not affect the results, as the distribution is symmetric (data not shown). We consider this novel result an important demonstration of the application of our analysis protocol; such a distribution has not been recorded previously for *R. sphaeroides*. Again, the methodological variants are all in close agreement. The distribution is unimodal, containing a single peak at the origin. We carried out a two-sided Kuiper test [44] on the *R. sphaeroides* dataset to compare the simulated distribution of inferred stopwise angles (shown in Figure 5(b)) with the experimentally-observed distribution. If these two distributions are similar, we are unable to determine whether the observed experimental distribution is significant, or whether it arises as a result of the bias inherent in our analysis method. Analysis of the experimental *R. sphaeroides* data indicates that 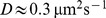 (see Figure S10 and Text S1); we use the conservative value 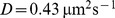 in our simulations. A two-sided Kuiper test reveals that the two distributions differ significantly (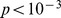, see Text S1 for details of the calculation). The result in Figure 8(b) is therefore more significant than the small bias introduced by the analysis methods, indicating that *R. sphaeroides* exhibit persistence over reorientation phases.

## Discussion

In this work we have demonstrated the effective application of novel analysis methods based on a modified HMM to tracking data acquired using a simple and relatively inexpensive experimental protocol. The result is a high-throughput method to characterise bacterial motion. We applied our methods to two species of bacteria that exhibit quite different motile behaviour and showed that we are able to estimate certain key distributions, such as the pdf of stopwise angle changes, plotted in Figures 8(b) and 8(c). This result has not been measured before in *R. sphaeroides*, and provides significant evidence that this bacterium exhibits persistence over reorientation events, which has important consequences for the modelling of their motion, and that of related flagellate bacteria. We note that persistence is a consequence of any reorientation process that occurs over a stochastic duration if some reorientation phases are sufficiently brief that the direction has not been fully randomised. Therefore, we propose that shorter reorientation events in the two species considered here lead to a greater degree of persistence. Testing this hypothesis is the topic of ongoing work.

The stopwise angle change distribution in *E. coli* (Figure 8(c)) has been measured previously by Berg and Brown [4] (see Figure 3 in that reference for comparison). In contrast with the bimodal distribution centred at approximately 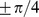 found in Berg and Brown's study, we find that the distributions in both *E. coli* and *R. sphaeroides* is unimodal and peaked about the origin. In addition, there is no significant difference between the distribution for these two species. For further comparison, Xie et al. measured the distribution of stopwise angle changes in *V. alginolyticus*, a bacterium that undergoes reversal events, and showed that the distribution is bimodal, with peaks at around 90 and 180 degrees [12]. The difference between the analysis methods used to extract stopping events in our study and that of Berg and Brown may provide an explanation for the discrepancy in our results. In the earlier study, a heuristic method is applied in which the framewise angle change must exceed 35 degrees for more than one frame to be labelled as a stop [4]. This may bias the analysis towards detecting stopping events with larger angle changes.

A further explanation for the discrepancy between this study and that of Berg and Brown may be the substantially different experimental protocols used in the two studies. Berg and Brown track individual bacteria at a frame rate of 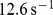, while we simultaneously track multiple bacteria at a frame rate of 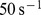. As a result, our datasets contain significantly more tracks: we analyse 1758 tracks in the *E. coli* wildtype dataset, compared with the 35 recorded by Berg and Brown [4]. Duffy and Ford [10] more recently used the same tracking apparatus to study *P. putida*, obtaining 80 tracks. However, the tracks we acquire have a lower mean duration: Berg and Brown [4] present a wildtype track 29.5 seconds in duration; by comparison the mean duration of our tracks is 1.5 seconds in the *R. sphaeroides* dataset and 6 seconds in the *E. coli* dataset. This difference in mean track duration is due to the lower magnification used in acquiring the *E. coli* dataset, in addition to the lower swimming speed of this species.

The duration of tracks is limited by the size of the focal plane and the fact that bacteria may swim out of focus, thus terminating the track. This reduction in track duration is a consequence of the high-throughput, unsupervised protocol used in this study, and is a limitation generally present in many recently-developed multiple cell tracking protocols [15], [29]. Whilst we obtain fewer measurements for each individual, we are able to measure significantly more robust population-wide statistics. As each cell is observed over a randomly-selected time interval in its lifetime, the shorter duration of the tracks has no consequences for our population measurements. Further work is required to determine whether shorter duration tracks reduce our ability to discern variations in the motile behaviour of an individual bacterium. By way of preliminary comment, we note that the appearance of the tracks with the longest duration (around 10 seconds) in the current dataset suggests that the motile behaviour observed in our tracks is not significantly different over a single order of magnitude of timescales. Furthermore, our approach is less subject to bias than a human-operated single-cell tracking protocol, as we image all cells within the field of view and discard tracks using a small number of well-justified censoring parameters. In contrast, any protocol in which the experimentalist may select which cells to track may be systematically biased in favour of a certain, idealised, type of motile behaviour.

A second novel contribution of the present work is the use of a systematic simulation study to validate our analysis methods and compare them with an established method. To our knowledge, no studies have previously compared analysis methods applicable to bacterial tracking data. The comparison indicates that the methods based on the HMM are significantly more robust to noise than the established heuristic method, generating significantly fewer FPs. Furthermore, the simulation study allowed us to determine the extent to which the results are biased by FPs (see Figure 5(b)). We used the results from our simulation study to show that the distribution of stopwise angle changes obtained from experimental data in *R. sphaeroides* (Figure 8(b)) differs from the distribution of FP stopwise angle changes obtained from simulated tracks (Figure 5(b)) with very high statistical significance. A quantification of the inherent bias in the analysis methodology has not been carried out in previous bacterial tracking studies [4], [45], thus it is unclear to what extent the statistics may be biased. We believe that our simulation approach therefore represents an important advance in the field of bacterial tracking.

An important caveat associated with the high-throughput tracking of many cells simultaneously is the inevitable presence of many tracks that do not appear to conform to the well-studied run-and-tumble model of motility. For example, a non-motile subpopulation has been observed in several similar studies [46]–[48]. Whilst these tracks may be of general interest, the present analysis methods are specifically developed to extract information about bacteria undergoing run-and-tumble motion, hence it is necessary to filter out incongruous tracks. In Materials and Methods, we have presented censoring approaches that mitigate such issues. In particular, the minimum bounding diameter and tortuosity are very useful characteristics for censoring tracks that might otherwise lead to spurious inferences. In particular, we discard the top 5% of tracks, ordered by tortuosity. This approach allows us to apply the same censoring method to multiple datasets without the need to specify multiple thresholds, and therefore permits unbiased comparisons to be made.

Manual inspection of the segmented tracks revealed a selection of tracks in which the new analysis methods appear to have performed well (see Figures 7 and S11). These tracks were manually selected from the dataset because they appear easy to interpret, with clear running and stopping phases. In addition, an example of a helical *R. sphaeroides* track was shown, for which both analysis methods clearly failed to infer the correct state sequence. The inclusion of post-processing helped to correct the inferred run probabilities.

The HMM approach takes advantage of the availability of non-motile and non-chemotactic mutant strains to obtain empirical prior information on the motion of the bacteria. Such strains are available for many bacterial species not considered in this study, for example *Campylobacter jejuni* [49], and *Caulobacter crescentus* [50]. The protocol developed is theoretically applicable to any bacterium that undergoes approximately discrete reorientation events of sufficient duration so as to be captured with a video microscope. It is encouraging that our analysis methods have proved applicable to two very different species of bacteria. There are substantial differences in the reorientation mechanisms of the two species: *E. coli* undergoes rapid, active reorientation, achieved by the displacement of individual flagellar helices from a peritrichous flagellar bundle, whereas *R. sphaeroides* reorientates more slowly, by halting the rotation of its single flagellum [5]. The mean stop duration parameter, 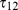, is larger in *R. sphaeroides*, as expected. Further work is required to determine whether all such bacteria are amenable to analysis in this way, however. For example, *Bacillus subtilis* is believed to accelerate into a running phase [51], which could contravene our two-state model of motion if the acceleration stage is long relative to the timescale of the microscopy.

A further possible application of the methods presented in this study is to the motion of certain eukaryotic species, such as the alga *Chlamydomonas*, which is known to exhibit motion that is superficially similar to the random swimming of bacteria [52]. However, this alga is approximately an order of magnitude larger than bacteria, and therefore exhibits significantly different properties, such as inertia and spatial sensing. Further work is needed to test whether our methods are applicable to such species.

The methods presented here may also be applied in situations where no mutant strains are available. The motion of non-motile bacteria may be reasonably approximated by a diffusive process, as is the case for the non-motile *R. sphaeroides* and *E. coli* in the present study [53]. Furthermore, it is possible to generate an estimate of the behaviour of bacteria in a running phase by manually selecting running phases in a wildtype dataset, although this is a subjective procedure that potentially biases the analysis. Whilst the present study concerns the analysis of a single, identified species of bacteria at any one time, there is also a demand to analyse samples containing multiple unknown bacterial species [54]. Further work is required to determine whether our analysis methods are applicable in these situations. For example, minor modifications should allow the HMM methods to be used to determine the likelihood that a given observed track arises from a reference model of motion.

The current experimental approach produces two-dimensional position coordinates for the cell centroids. We have therefore implicitly projected the true three-dimensional motion of the bacteria swimming in the bulk onto the microscope's image plane. Hill and Häder [55] analysed the effect of projection of tracks onto a two-dimensional plane and concluded that, for their purposes, the error introduced in the observed mean speed is small (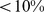). The authors assume an infinite focal depth for their calculation, whereas the focal depth in our setup is small compared to the dimensions of the image plane. We therefore expect the errors caused by projection in our case to be substantially smaller. A further consequence of performing tracking away from a surface within a single focal plane is that bacteria may freely swim out of focus, causing the track to be terminated and leading to tracks of relatively short duration [17]. It is possible to track bacteria in three dimensions, and several groups have made use of various three-dimensional tracking methods to investigate bacterial swimming [4], [10], [28], [29], [45], [56], [57]. The process for obtaining three-dimensional tracks is, however, generally more complex than the method we use and in many cases this leads to a reduced number of tracks available for analysis. Digital holographic microscopy is a promising recent development that could potentially allow the tracking of multiple bacteria simultaneously in three dimensions in a fixed field of view [58]. The HMM-based approaches presented here can be extended in a straightforward manner to deal with three-dimensional data.

Software implementing the methods described in this study is provided in the supporting file Software S1. It is fully documented and written in Python to make it compatible with all major operating systems. The applications of the analysis methods presented here are of potential benefit in a wide variety of bacterial research, including studies of pathogenicity, biofilm formation, and the response of bacteria to chemoattractants and changing environments. In particular, the field of microfluidics is a promising area for further development, as it allows the tracking of bacteria in a well-defined concentration gradient of chemoattractant, as demonstrated by Ahmed and Stocker [17]. In this case, a modification would be required to incorporate the spatial variation of the transition matrix 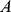, reflecting the heterogeneous chemoattractant concentration. The ability to quickly assess and compare the motility of a variety of related bacterial strains, or different species, is a powerful addition to the methodological toolbox of the bacteriologist.

## Materials and Methods

### Acquisition of bacterial tracks

Imaging and tracking was performed on three different strains of *R. sphaeroides*: wildtype (WS8N), a non-motile mutant (JPA467) and a non-chemotactic mutant that is incapable of stopping (JPA1353). Details of the experimental protocol used to create the mutant strains, and the growth conditions, are given in [5]. Some typical raw footage of *R. sphaeroides* is provided in Video S1. Three strains of *E. coli* were also used: wildtype (RP437), non-motile (CheY^**^), and non-chemotactic (ΔCheY). Bacteria were imaged in a homogeneous solution of motility buffer using a tunnel slide. Imaging was performed at 50 frames per second using a Nikon phase contrast microscope with a 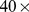 magnification objective lens in the case of *R. sphaeroides* and a 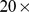 objective in the case of *E. coli*. The images are captured in 256 level greyscale, 640 pixels (px) wide and 480 px in height, equivalent to 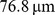 wide and 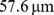 high in the case of *R. sphaeroides* and twice those dimensions for *E. coli*. For comparison, a typical *R. sphaeroides* cell is approximately ellipsoidal, with axial and equatorial diameters of around 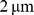 and 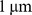, respectively. Imaging was performed with the microscope focused approximately 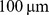 below the top coverslip, and at least this distance from the bottom surface of the microscope slide. This is sufficiently far from either surface that we may neglect surface effects, which are known to cause bacteria to swim in arcing trajectories [45] The observed cells are swimming freely in the medium and may stray out of the focal plane. Typically between 10 and 20 minutes of footage are acquired for each strain, from each of which we obtain between 3000 and 7000 tracks. The tracking procedure is able to cope with a large variation in the density of cells within the field of view, and this value changes depending on the level of magnification used. We typically aimed for around 20–40 cells visible within the field of view in the case of 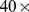 magnification, and 50–80 cells in the case of 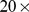 magnification. Both magnification levels used provided sufficient spatial resolution to find centroids with acceptable accuracy. Further work is necessary to determine whether even lower levels of magnification would allow us to increase the throughput of the experiment without compromising on accuracy. The frame rate of the camera should be sufficiently rapid that reorientation events can be imaged, and preferably so that most events last for greater than a single frame.

We performed cell tracking in two stages. First, in the object detection stage, each frame in a video was processed to establish the centroids of each visible cell. Second, in the data association stage, centroids in each frame were connected to form tracks. The object detection stage is carried out in several steps:

1. compute the background value of each pixel as its mean intensity over all frames;
2. subtract the background intensity from all frames;
3. find pixels in each frame with intensities after background subtraction above the threshold value 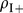 and below the threshold value 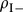;
4. cluster groups of pixels that are 4-connected, meaning that every pixel in a cluster has another pixel in the same cluster in one of the four neighbouring sites around it;
5. discard any clusters containing fewer than a defined number of pixels, 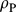;
6. find the centroid (centre of mass) of each of the remaining clusters.

The centroids computed using this method represent the targets present in each frame. The initial background subtraction ensures that any static image artefacts, such as dust on the microscope lens or impurities stuck to the coverslip, are removed from the video. The parameters 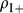 and 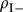 were selected separately for each video based on manual verification that the process correctly segmented cells in the images. The values of these parameters were chosen to minimise the number of missed detections, at the expense of producing additional FPs, as the data association routine is robust to high levels of FPs [27]. The minimum cluster size constraint was applied to the region data to remove spurious targets, which are too small to be cells. The minimum cluster size was fixed at 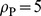 px, which is substantially below the mean cross-sectional area of a cell. This resulted in the removal of a significant number of FPs whilst having no effect on true positives. Some errors arise in the process of computing the cell centroid, due to the relatively low contrast of the microscope images. We estimate that such errors should be no greater than half the diameter of a cell body. In order to manually confirm that cell centroid calculation is sufficiently robust for our purposes, tracks from non-chemotactic cells were examined to ensure that they mainly showed smooth swimming, with no overly jagged sections. A further consequence of the low contrast images is that it is not possible to determine cell orientation on this scale; this parameter must therefore be inferred from the angle change between each triplet of consecutive centroids.

The data association method used in this study is a multitarget tracking scheme based on the probability hypothesis density filter. We use an implementation described in [27], which has been applied to microscope videos similar to those used in this study. Video S2 shows the raw microscopy footage of *R. sphaeroides* overlaid with tracks. As described in *Analysis methods*, the tracker performs less well when cells are in a stopped phase, as the errors in centroid detection are more significant. Manual inspection of tracks shows well-defined stopping phases in the wildtype strains, however the apparent trajectory during a stop is not accurate. This provides the basis for the modification to the HMM, discussed in the section *Hidden Markov model methods*.

### Bootstrapping method for estimation of transition probability confidence intervals

When optimising the value of the transition parameters 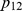 and 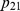, we require an estimate of the uncertainty in our final MLE. This is achieved using simple bootstrapping [37], in which we resample the tracking dataset by drawing the same number of tracks randomly with replacement. The optimisation procedure is then repeated on the new selected dataset, to achieve a new MLE. This process is repeated for 1000 iterations, after which we sort the bootstrapped MLE transition parameters. We finally use the 2.5th and 97.5th percentile values from the sorted list of 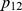 and 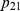 as estimates of the confidence interval.

### Censoring tracking datasets

Preliminary scrutinisation of our *R. sphaeroides* and *E. coli* tracking data reveals that a significant proportion of tracks that do not appear to be well described by the run-and-tumble motility described in previous studies [4], [59]. These tracks are either very jagged in their appearance, exhibit unrealistically large movements between frames, or seem to arise from a diffusing object, rather than an actively swimming cell. Possible causes of such tracks include errors in the tracking process, non-motile bacteria, and bacteria with defective motility apparatus. First, the process used to extract tracks from microscope videos may occasionally produce a failed track, for example by linking the trajectories of two different cells, or incorporating a false detection into the trajectory. This is a particular concern if the failed track displays behaviour that differs substantially from the true motion of the observed bacteria, since even a small number of failed tracks may dramatically affect the inferences that are drawn. In order to avoid this issue, tracks containing one or more framewise speeds greater than a threshold value, denoted 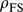, are considered to be anomalous and discarded from the dataset. The value of 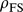 is determined by considering the observed distribution of framewise speeds in the non-chemotactic strain; this gives an indication of the range of speeds exhibited. An upper threshold is then selected that causes outliers to be discarded. In the case of *R. sphaeroides*, whose mean swimming speed is approximately 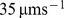, we select 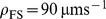. The mean swimming speed of *E. coli* is 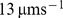 and we choose 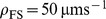. In both cases, 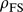 is significantly greater than the mean swimming speed. We allow such a large margin for variation in the framewise speed as small errors in consecutive frames can generate large fluctuations in the apparent framewise speed. We do not wish to discard tracks containing a few instances of such inaccuracies, since these quantities will not dominate the population average. This effect is expected to be minor when all tracks in a dataset are considered, and we note that over- and underestimation of the framewise speed are equally probable. Observed framewise speeds above the cutoff value of 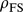 are unlikely to arise from such a source of noise; these are instead treated as a tracking error and the whole track is discarded.

**Figure 9:**
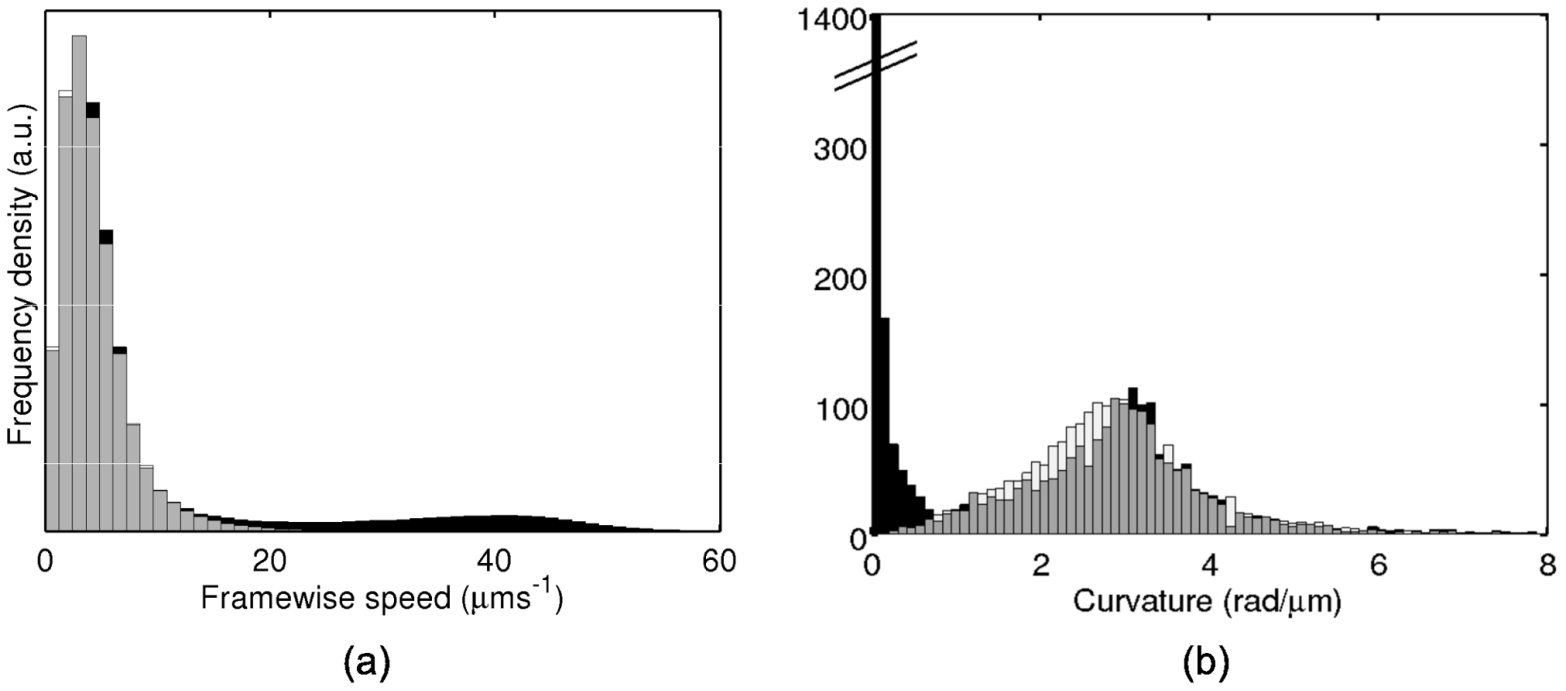
Motile characteristics extracted from the non-motile and non-chemotactic *R. sphaeroides* tracking datasets. (a) Histogram of framewise speeds for the non-chemotactic (black bars) and non-motile (white bars) datasets. Overlapping regions are shown in grey. The distributions have been scaled so their maxima coincide. (b) Histogram of median curvature (defined below in equation (13) ) computed for all tracks in the non-chemotactic (black bars) and non-motile (white bars) datasets. Intersecting regions are shown in grey. Note that the 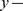
axis is broken; the density at low curvatures dominates the non-chemotactic histogram. The datasets have been censored to remove failed tracks (see text for details).

In addition to tracker errors, a second consideration is the presence of a significant portion of non-motile tracked cells, as is usually observed in experiments of this kind [46]–[48]. Reasons for a lack of motility include cell death, a defective component in the cellular motility machinery, and cell damage due to experimental handling. Figure 9 provides evidence for the presence of a non-motile subpopulation in the non-chemotactic *R. sphaeroides* strain by comparison with the non-motile strain. As Figure 9(a) demonstrates, the observed distribution of framewise speeds for the non-chemotactic strain is bimodal, with a peak at low speeds that overlaps almost exactly with the non-motile distribution. This suggests that the low speed subpopulation in the non-chemotactic strain is due to non-motile cells. Similarly, in Figure 9(b), non-chemotactic *R. sphaeroides* bacteria exhibit a bimodal distribution of median curvatures. The subpopulation with higher median curvatures corresponds very closely to the non-motile population.

A third way in which the experimental data differ from the simulated data is the wide range of tortuosities exhibited by real tracks, due to variation within the populations of bacteria being studied. Several tracks appear to be highly tortuous, possibly as a result of bacteria swimming in severely helical paths or with substantial cell body motion. Possible causes for tortuous tracks include damaged or defective flagella, and two bacterial cells swimming whilst stuck together, prior to cell division. None of the analysis methods discussed herein are able to cope with highly tortuous tracks, as these exhibit many large framewise angle changes and low framewise speeds in the running phase. It is therefore challenging to discern stopping phases in such tracks, either automatically or by manual inspection. Tortuous tracks are apparent in the non-chemotactic and wildtype datasets and it is necessary to remove them from the dataset before performing any further analysis.

Our approach to censoring tracks is based on a two-variable representation of a track used by Miño et al. [47]. Each track is summarised in terms of the mean absolute framewise angle change (MAC), and the normalised effective mean speed (NEMS). The NEMS is defined as the ratio of the effective mean speed (EMS) to the mean framewise speed. The EMS is in turn given by the diameter of the smallest circle that encloses the entire track (denoted the minimum bounding diameter, MBD) divided by the total duration of the track. Thus the NEMS takes values between zero and one, and quantifies how straight the track is, with one interpreted as a line that doesn't deviate from a straight path and smaller values indicating increasingly undirected motion.

**Figure 10:**
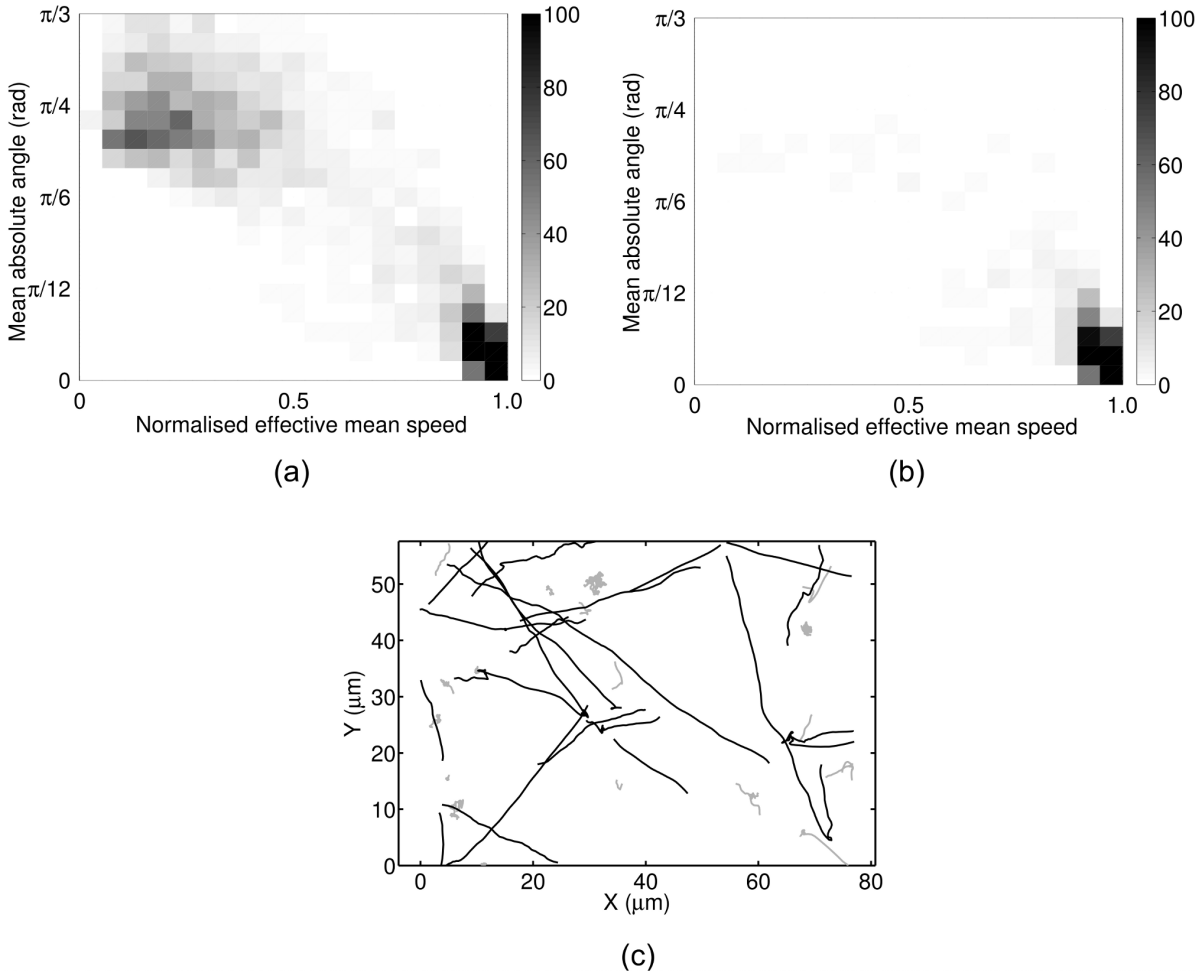
Results illustrating the censoring process in *R. sphaeroides*. (a) MAC-NEMS plot for the non-chemotactic dataset, before censoring. (b) MAC-NEMS plot for the non-chemotactic dataset, after censoring. (c) A random selection of 40 tracks from the wildtype dataset, with censored tracks shown in grey and remaining tracks shown in black.

Miño et al. note that a population consisting of self-propelled particles (which is a good model for motile bacteria) and non-motile diffusing particles exhibits a well-separated bimodal distribution in the MAC-NEMS plot [47]. Figure 10(a) shows such a plot for the non-chemotactic strain of *R. sphaeroides*, before any censoring. Two modes are clearly visible, one with high MAC and low NEMS corresponding to non-motile cells, and one with low MAC and high NEMS corresponding to motile cells. We use this representation of tracks to determine the effectiveness of our censoring approach.

We also require a measure of the tortuosity of a track, as this is a useful property for the purposes of filtering the dataset. Several methods have been proposed for estimating tortuosity [60]; we employ a method proposed by Lewiner et al., in which a three-point estimator of the curvature of a track is used as a measure of the tortuosity [61]. The curvature is defined for a given position, 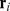, 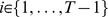, in a track by
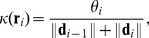
(13)where the notation is introduced in the Results section and illustrated in Figure 1. The curvature is undefined for the first and last points in a track, as we require three adjacent points to estimate it. We use the median value of the absolute curvature of a track as a summary statistic, as this has been used previously to characterise trajectories [20].

The non-motile tracks are not censored beyond the application of the threshold 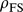, as any further censoring would remove all of the remaining tracks. For the non-chemotactic and wildtpe strains we censor tracks in two stages. We first filter out non-motile tracks by imposing a minimum value of 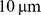 for the MBD, and discard tracks whose MBD is lower than this cutoff value. This ensures that tracks that do not cover a sufficiently large region of the field of view are removed from the dataset; in practice, tracks that do not meet this threshold are non-motile or of very short duration. Finally, the top five percent of tracks, ordered by median curvature, are discarded, following Alon et al. [18]. This stage is necessary to remove the remaining non-motile and anomalously tortuous tracks. Discarding an arbitrary proportion of tracks may lead to anomalous tracks remaining in the dataset, or tracks of interest being removed. Nonetheless, this approach has the advantage that the same parameters may be used to censor a wide range of datasets. In this study, for example, we use the same censoring parameters to remove defective tracks from both *R. sphaeroides* and *E. coli* tracking data.

Figure 10(b) shows the MAC-NEMS plot for the non-chemotactic *R. sphaeroides* strain following censoring. The density at high MAC has been filtered out, leaving mainly tracks that lie in the correct region of the plot corresponsing to motile cells. Similar plots for wildtype *R. sphaeroides* and both non-chemotactic and wildtype *E. coli* are shown in Figures S7, S8, S9; in all cases, the censoring process removes tracks that lie in the high MAC, low NEMS region.

The number of tracks in each of the datasets before and after the censoring stages is given in Table 2. The censoring stage removes a large proportion of the initial tracks, with most failing on the minimum MBD criterion. This is an important stage of the analysis process, as most of these tracks are due to non-motile cells or very short duration tracks, neither of which are desirable in the final dataset. Figure 10(c) shows a representative sample of tracks before and after the censoring process. The dataset initially contains a large proportion of tracks from non-motile or motility-defective bacteria. After censoring, these tracks have been removed, whilst still retaining longer tracks that exhibit stops.

**Table 2:** Effect of censoring the datasets.

Dataset	*Rs* nm	*Rs* nc	*Rs* wt	*Ec* nm	*Ec* nc	*Ec* wt
**Initial number tracks**	5627	3773	6832	3669	3562	5757
**Number above** *ρ*_FS_	47	212	706	500	492	979
**Number below minimum MBD**	-	1859	2928	-	1219	2811
**5% removed by median curvature**	-	86	160	-	93	99
**Number remaining**	5580	1616	3038	3169	1758	1868

The number of tracks in each of the datasets considered, before and after censoring. *Rs* denotes *R. sphaeroides*, *Ec* is *E. coli*, nm is non-motile, nc is non-chemotactic, wt is wildtype. Dashes indicate that a stage of the censoring is not applicable.

### Post-processing

Post-processing is implemented as follows:

1. find the duration of all inferred running phases;
2. convert all running phases with duration less than 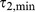 to stops;
3. recalculate to find the duration of all stopping phases;
4. convert all stopping phases with duration less than 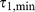 to runs.

The process is illustrated in Figure 3, in which the short stop inferred at around 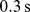 is removed by the application of post-processing. The relabelling of short runs before short stops introduces a bias towards stops when sustained rapid oscillations occur between the two states (the short run sections will first be converted to stops, resulting in a larger stopped section). We choose to proceed in this fashion as we place greater importance on identifying every stop, possibly at the expense of including some false positives or inferring overly long stopping phases.

## Supporting Information

Figure S1 **Joint and marginal estimated observation pdfs for the non-chemotactic strain of** ***R. sphaeroides*** **.**
(EPS)

Figure S2 **Joint and marginal estimated observation pdfs for the non-motile strain of** ***R. sphaeroides*** **.**
(EPS)

Figure S3 **Joint and marginal estimated observation pdfs for the non-chemotactic strain of** ***R. sphaeroides*** **, rescaled to show noise.**
(EPS)

Figure S4 **Joint and marginal estimated observation pdfs for the non-motile strain of** ***R. sphaeroides*** **, rescaled to show noise.**
(EPS)

Figure S5
**Plot showing simulated tracks with varying levels of added noise.** (a) 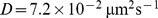, (b) 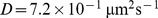, (c) 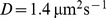, (d) 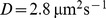.(EPS)

Figure S6
**Observed distributions extracted from simulated non-motile (white bars) and non-chemotactic (black bars) tracks.** Grey bars denote overlapping regions. Noise is applied with 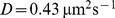. (a) Framewise speeds. (b) Framewise angle changes.(EPS)

Figure S7 **The negative log likelihood surface for a simulated non-chemotactic dataset.**
(EPS)

Figure S8 **MAC-NEMS plots for wildtype** ***R. sphaeroides*** **before (a) and after (b) censoring.**
(EPS)

Figure S9 **MAC-NEMS plots for non-chemotactic** ***E. coli*** **before (a) and after (b) censoring.**
(EPS)

Figure S10 **MAC-NEMS plots for wildtype** ***E. coli*** **before before (a) and after (b) censoring.**
(EPS)

Figure S11
**Estimation of the level of noise in the experimental data.** Mean squared displacement of the non-motile *R. sphaeroides* dataset (solid line), overlaid with a linear fit to the data from time 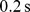 onwards (dashed line). The gradient of the dashed line is approximately 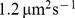.(EPS)

Figure S12
**Manual inspection of wildtype** ***E. coli*** **tracks, analysed with the speed-only HMM method.** Tracks appear similar when the full method is used (data not shown). Green indicates a running phase, red indicates a stopping phase, small circles indicate the starting position of the track, and pairs of arrows show the direction of travel of the bacterium immediately prior to and after a stop. The top plot shows tracks where the methods appear to have performed well. The lower plot shows tracks for which the state sequence shows very many transitions over the course of each track; these appear to arise from highly tumbly swimmers, and are likely to be among the most tortuous tracks remaining in the dataset following the censoring approach. All tracks are plotted on the same scale; the plot is approximately 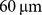 wide.(EPS)

Software S1 **Zipped folder containing Python implementation of the analysis method described in this study, together with experimental data.** Details for how to install the software is given in the doc subfolder, while a thoroughly documented example of how to run the code is provided in the file usage_example.py. Further documentation of this software is provided online at http://www.2020science.net/software/bacterial-motility-analysis-tool.
(ZIP)

Text S1 **Supplementary text providing further details on: assessing whether our analysis methods may be blindly applied to tracks from a movement model that does not fit the two-state model assumed for our HMM method; estimating the level of noise present in our experimental data; and testing for statistical significance of the observed stopwise angle change distribution.**
(PDF)

Video S1 **Short clip of the raw microscopy video data obtained for wildtype** ***R. sphaeroides*** **.** Details of the experimental protocol are provided in the main text and [5].
(AVI)

Video S2 **The same footage presented in Video S1, but overlaid with cell tracks obtained using a multitarget tracking scheme based on the probability hypothesis density filter.** The implementation of this scheme are described in [27].
(MPG)
