# Supplementary material for: Novel Methods for Analysing Bacterial Tracks Reveal Persistence in Rhodobacter sphaeroides
Source: PLoS Comput Biol. 2013 Oct 24;9(10):e1003276. doi: 10.1371/journal.pcbi.1003276 (PMC3812076; doi:10.1371/journal.pcbi.1003276)
Supplement: Text S1 — Supplementary text providing further details on: assessing whether our analysis methods may be blindly applied to tracks from a movement model that does not fit the two-state model assumed for our HMM method; estimating the level of noise present in our experimental data; and testing for statistical significance of the observed stopwise angle change distribution. (PDF) [file pcbi.1003276.s014.pdf]

Novel methods for analysing bacterial tracks reveal persistence in *Rhodobacter sphaeroides*: Supporting Information Text S1

Attempting to analyse tracks from an incompatible movement model

In order to check whether our analysis methods may be blindly applied to tracks from a movement model that does not fit the two-state model assumed for the HMM methods, we attempt to analyse a simulated non-chemotactic dataset instead of a simulated wildtype dataset. In such a scenario, the tracks being analysed contain no reorientation events. Figure S6 shows the negative log likelihood surface computed for the non-chemotactic dataset using the full HMM method. As expected, it is independent of the parameter p_{01}, as the probability of a transition from a stop to a run is irrelevant in the absence of stopping phases. Attempting to find the maximum likelihood estimate of the transition parameters by minimising the negative log likelihood fails in this case. This result demonstrates that our HMM analysis methods contain a built-in validity check, though further work is required to determine whether other incompatible models would raise such an error.

Estimating the level of noise in the experimental data

The level of noise included in our computational simulations is denoted D. Since we use Gaussian noise in the simulations, the noise is equivalent to translational Brownian motion, and D is the translational diffusion coefficient. We may therefore estimate D from our non-motile dataset by fitting the mean squared displacement (MSD) with a linear expression, the slope of which is equal to $4D$ for two-dimensional data [1]. Figure S10 shows the MSD observed in the non-motile strain, overlaid with a linear fit. The MSD is not well described by a linear fit for the first 0.2 s, so we use the remaining data for the fit, which gives an estimated value $D \approx 0.3\ \mu\mathrm{m}^2\mathrm{s}^{-1}$.

Two-sided Kuiper test for the significance of the observed stopwise angle change distribution

The two-sided Kuiper test is a statistical method of assessing the null hypothesis that two sets of circular random variables are drawn from the same underlying distribution [2]. In our specific example, we require to test whether the experimentally-observed stopwise angle changes (Figure 8(b)) may be drawn from the inferred stopwise angle changes in the simulated data with an underlying uniform distribution (Figure 5(b)). Figure 5(b) departs from uniform due to the presence of false positives, hence any statistical similarity between this distribution and that observed experimentally would indicate that the experimental results may arise from bias at the analysis stage, as opposed to being biologically meaningful. The test and its implementation in MATLAB is described in [3].

Bibliography

1. Das R, Cairo CW, Coombs D (2009) A hidden Markov model for single particle tracks quantifies dynamic interactions between LFA-1 and the actin cytoskeleton. PLoS Comput Biol 5: e1000556+.

2. Kuiper NH (1962) Tests concerning random points on a circle. Nederl Akad Wetensch Proc Ser A 63: 38–47.

3. Berens P (2009) CircStat: A MATLAB toolbox for circular statistics. J Stat Software 31: 1–21.
